# Antibacterial, antifungal, and antiviral effects of three essential oil blends

**DOI:** 10.1002/mbo3.459

**Published:** 2017-03-14

**Authors:** Amandine Brochot, Angèle Guilbot, Laïla Haddioui, Christine Roques

**Affiliations:** ^1^ PiLeJe Paris Cedex 15 France; ^2^ Fonderephar Toulouse Cedex 09 France; ^3^ Laboratoire de Génie Chimique UMR 5503 Faculté des Sciences Pharmaceutiques Université Paul Sabatier Toulouse France

**Keywords:** antimicrobials, *E. coli*, Fungi, infection, viruses

## Abstract

New agents that are effective against common pathogens are needed particularly for those resistant to conventional antimicrobial agents. Essential oils (EOs) are known for their antimicrobial activity. Using the broth microdilution method, we showed that (1) two unique blends of *Cinnamomum zeylanicum*,* Daucus carota*,* Eucalyptus globulus* and *Rosmarinus officinalis* EOs (AB1 and AB2; cinnamon EOs from two different suppliers) were active against the fourteen Gram‐positive and ‐negative bacteria strains tested, including some antibiotic‐resistant strains. Minimal inhibitory concentrations (MICs) ranged from 0.01% to 3% v/v with minimal bactericidal concentrations from <0.01% to 6.00% v/v; (2) a blend of *Cinnamomum zeylanicum*,* Daucus carota, Syzygium aromaticum, Origanum vulgare* EOs was antifungal to the six *Candida* strains tested, with MICs ranging from 0.01% to 0.05% v/v with minimal fungicidal concentrations from 0.02% to 0.05% v/v. Blend AB1 was also effective against H1N1 and HSV1 viruses. With this dual activity, against H1N1 and against *S. aureus* and *S. pneumoniae* notably, AB1 may be interesting to treat influenza and postinfluenza bacterial pneumonia infections. These blends could be very useful in clinical practice to combat common infections including those caused by microorganisms resistant to antimicrobial drugs.

## 1 Introduction

Antimicrobial resistance poses a serious threat to the effective treatment of an ever‐increasing range of infections caused by bacteria, fungi and viruses. Worldwide, antibiotic resistance is increasing. For example, *Escherichia coli*,* Klebsiella pneumoniae*,* Streptococcus pneumoniae* have reported reduced antibiotic susceptibility, which exceeded 50% in most countries that provided data to the WHO Antimicrobial Resistance Global Report on Surveillance (WHO, 2014). Candidiasis has also become substantially problematic, with *Candida albicans* showing increased resistance to common antifungal agents (Goncalves, Souza, Chowdhary, Meis, & Colombo, 2016; Hawser & Douglas, 1995). The recent pandemic of a novel H1N1 influenza viral strain and emerging strains resistant to commonly used anti‐herpes simplex drugs also emphasizes the need to identify effective approaches to prevent and treat viral infections (Boivin, 2013; James & Prichard, 2014).

This increasing resistance has created a need to develop new antimicrobial agents. Essential oils (EOs) are good candidates as studies have shown that individual EOs and their isolated compounds, including terpenes and terpenoids (1,8‐cineole, carvacrol) and aromatic compounds (cinnamaldehyde and eugenol) have antimicrobial activity against a wide range of pathogens, with various spectrums of activity (Bassole & Juliani, 2012; Friedman, Henika, & Mandrell, 2002; Jantan, Karim Moharam, Santhanam, & Jamal, 2008). The antimicrobial effects of EOs are linked to their composition and cytotoxic effects, which cause cell membrane damage. EO compounds are lipophilic, and so pass through the cell wall and cytoplasmic membrane. They disrupt the structure of the polysaccharide, fatty acid, and phospholipid layers, making the membrane permeable (Bakkali, Averbeck, Averbeck, & Idaomar, 2008). Unfortunately, EOs do not specifically target pathogens; they can also affect eukaryotic cells in a reversible or irreversible manner (Carson, Hammer, & Riley, 2006). In extreme cases, EO cytotoxicity can lead to apoptosis, necrosis, and organ failure (Tisserand & Young, 2013). Therefore, EOs have to be used carefully, within the daily intake limits defined by the relevant authorities when available (EMEA and HMPC 2010, 2011; FAO and WHO 2003).

Three different EO blends were formulated, taking into account the specific activity of each. The first two (AB1 and AB2) contained EOs from *Cinnamomum zeylanicum*,* Daucus carota*,* Eucalyptus globulus*, and *Rosmarinus officinalis*, which differed only in that the cinnamon EOs were provided by two different suppliers. These EOs were selected for their antibacterial effects that had been observed, either individually or in pairs, in previously published studies (for review see Bassole & Juliani, 2012). *Eucalyptus globulus* and *Cinnamomum Zeylanicum* EOs also have been reported to have antiviral activity (Astani, Reichling, & Schnitzler, 2010; Cermelli, Fabio, Fabio, & Quaglio, 2008; Vimalanathan & Hudson, 2014). The third blend (AF) contained EOs from *Cinnamomum zeylanicum*,* Daucus carota*,* Syzygium aromaticum*,* Origanum vulgare*, which are known for their antifungal activity (Khan & Ahmad, 2011; Pinto, Vale‐Silva, Cavaleiro, & Salgueiro, 2009; Tavares et al., 2008; Zore, Thakre, Jadhav, & Karuppayil, 2011).

The antibacterial activity of AB1 and AB2 was evaluated in vitro against a selection of Gram‐positive and Gram‐negative bacteria, with or without antibiotic resistance, AB1 was evaluated for antiviral activity and AF was assessed for activity against different *Candida* strains.

## 2 Materials and Methods

### 2.1 Essential oil blends

Blends AB1 and AB2 were composed of equal parts (3.52% each) of *Eucalyptus globulus* CT cineol (leaf) and *Cinnamomum zeylanicum* CT cinnamaldehyde (bark), 3.00% of *Rosmarinus officinalis* CT cineol (leaf), 1.04% of *Daucus carota* CT carotol (seed), and 88.90% of *Camelina sativa* oil (seed).

Blend AF contained equal parts (3.53% each) of *Cinnamomum zeylanicum* CT cinnamaldehyde (bark), *Syzygium aromaticum* CT eugenol (Synonymous: *Eugenia caryophyllus* Sprengel, cloves), and *Origanum vulgare* CT carvacol (aerial parts), 1.04% of *Daucus carota* CT carotol (seed), and 88.35% of *Camelina sativa* oil (seed).

All EOs were provided by Golgemma (Espéraza, France) except for *C. zeylanicum* in AB1 which was from Bontoux (Saint‐Aubin‐sur‐l'Ouvèze, France). *Camelina sativa* oil was provided by Polaris (Pleuven, France). Blends were stored at 4°C until used.

The EO extraction method and composition are provided as Supporting information.

### 2.2 Bacterial and fungal strains

Fourteen bacterial strains from the American Type Culture Collection (ATCC; Molsheim, France), the Pasteur Institute Collection (CIP; Paris, France), or from clinical samples (*Escherichia coli* UTI89 and extended‐spectrum beta‐lactamase positive [ESBL]) were tested. There were four Gram‐positive strains: *Streptococcus pyogenes* CIP 5641T, *Streptococcus pneumoniae* CIP 104471, *Listeria monocytogenes* CIP 82110T, and *Staphylococcus aureus* MRSA ATCC 33591; and ten Gram‐negative strains: *Pseudomonas aeruginosa* CIP 103467, *Proteus mirabilis* CIP 103181T, *Escherichia coli* ESBL, *Escherichia coli* UTI 89, *Klebsiella pneumoniae* CIP 8291T, *Salmonella typhimurium* CIP 6062T, *Yersinia enterocolitica* CIP 8027T, *Bacteriodes fragilis* ATCC 25285, *Haemophilus influenza* IP 102514, and *Branhamella catarrhalis* CIP 7321T. *Pseudomonas aeruginosa* CIP 103467, *Staphylococcus aureus* MRSA ATCC 3359, and *Escherichia coli* ESBL were selected for their marked natural or acquired resistance to antibiotics.

The following six fungal strains were tested as follows: *Candida albicans* DSM 1386, *Candida glabatra* DSM 11226, *Candida tropicalis* IP 2148.93, *Candida albicans* F26, *Candida albicans* F35, and *Candida albicans* F78. Two were from Deutsche Sammlung von Mikrooganismen und Zellkulturen (DSM), one from the Pasteur Institute (IP), and three were clinical isolates (F).

### 2.3 Antibacterial and antifungal assays

Strains were preserved at −80°C and subcultured on (1) trypcase soy agar (Biomérieux, Craponne, France) under aerobic conditions at 36°C for *P. aeruginosa* CIP 103467, *P. mirabilis* CIP 103181T, *E. coli* UTI 89, *S. thyphimurium* CIP 6062T, *Y. enterocolitica* CIP 8027T, *K. pneumoniae* CIP 8291T, *S. aureus* MRSA ATCC 33591, and *E. coli* ESBL; (2) Columbia agar with 5% sheep erythrocytes (Biomérieux) under CO_2_ or anaerobic conditions at 36°C for *S. pyogenes* CIP 5641T, *S. pneumoniae* CIP 104471, *L. monocytogenes* CIP82110T, *B. fragilis* ATCC 25285, *H. influenza* IP 102514 and *B. catarrhalis* CIP 7321T; and (3) Sabouraud agar (Biomérieux) under aerobic conditions at 30°C for yeasts. Suspensions were prepared in sterile distilled water to obtain a final inoculum of 10^8^ CFU*/*ml for bacteria and 10^7^ CFU*/*ml for yeasts.

Blends AB1 and AB2 were tested for their antibacterial activity and AF for its antifungal activity according to a previously reported micromethod (Ibrahim et al., 2012). Tests were also performed with amoxicillin for bacteria and amphotericin B for yeasts as a control for microorganism sensitivity.

Each blend was diluted, using twofold steps in microtiter plates in culture medium: (1) Muller Hinton (MH) broth (Biomérieux) for *P. mirabilis* CIP 103181T, *E. coli* UTI 89, *S. typhimurium* CIP 6062T, *Y. enterocolitica* CIP 8027T, *K. pneumoniae* CIP 8291T, *S. aureus* MRSA ATCC 33591, *P. aeruginosa* CIP 103467, and *E. coli* ESBL; (2) MH broth supplemented with 10% fetal calf serum (PAN‐Dutscher) under CO_2_ or anaerobic conditions at 36°C for *S. pyogenes* CIP 5641T, *S. pneumoniae* CIP 104471, *L. monocytogenes* CIP 82110T, *B. fragilis* ATCC 25285, and *B. catarrhalis* CIP 7321T; (3) MH broth supplemented with 10% fetal calf serum (PAN‐Dutscher) and 1% Polyvitex (Biomérieux) for *H. influenza* IP 102514; and (4) Sabouraud (Biomérieux) for yeasts, from column 1 to column 10. Columns 11 and 12 were maintained for sterility control (without product or microorganisms) and growth control (without product and with microorganisms). The twofold dilutions led to emulsions allowing the conduct of tests. Inoculation was performed, using a multipoint inoculator (Denley) under a volume of approximately 1.5 μl for each suspension and microplates were incubated as described above.

Minimal inhibitory concentration (MIC) was defined as the concentration of test compound at which no macroscopic sign of cellular growth was detected in comparison to the control without compound. It was determined for bacteria after incubation at 36°C for 24 hr and yeasts at 30°C for 24 hr in the presence of serial dilutions of the test compounds. Minimal germicidal concentrations for bacteria (MBC) or fungi (MFC) was defined as the concentration of compound at which no macroscopic sign of cellular growth was detected compared to the control upon subculturing. These concentrations were determined by subcultivating on corresponding agar plates (MH agar or supplemented MH or Sabouraud agar) after incubating bacterial and fungal strains.

All experiments were performed in duplicate at each concentration, using a micromethod analysis based on the CA‐SFM guidelines.

### 2.4 Viral strains and antiviral activity

Antiviral activity of AB1 was tested with influenza A H1N1 ATCC VR‐R 1520 and oral herpes simplex HSV1 ATCC VR‐1383. Tests were performed according to NF EN 14476 (AFNOR 2015). The H1N1 strain was amplified on MDCK cells (CCL‐34, ATCC) and HSV1 on VERO cells in EMEM medium (PAN‐Dutscher). Virus suspension was added to the test compound with interfering substance under clean conditions (1% PBS, Sigma Aldrich). This mixture was maintained at 35°C ± 1 for 60 min ± 10. The activity was stopped by the molecular sieving method, using a sieve filter (Sephadex LH 20). Neutralization of the product was validated by passing it through Sephadex at a dilution 1/10.

Virus titration on cells in suspension was performed in microplates. A dilution series with a factor of four was prepared in an ice‐cold medium for 30 min in glass tubes. The dilution was then transferred into microtiter plates before the cell suspension was added in each well. Viral cytopathic effect was read under an inverted microscope after 4 days of incubation and determined by the Spearman–Kärber method (Lorenz & Bogel, 1973) according to the following formula:

Negative logarithm of 50% end point = negative logarithm of the highest virus concentration used – ([Sum of % affected at each dilution/100 ‐ 0.5] X [log of dilution])

Reduction in virus infectivity was calculated from the difference of log virus titers before and after treatment. The product was considered to be virucidal when log reduction was ≥4.

## 3 Results and Discussion

### 3.1 Antibacterial activity of AB1 and AB2

Blends AB1 and AB2 exhibited both bacteriostatic and bactericidal effects against all Gram‐positive and Gram‐negative bacteria tested, with MICs ranging from 0.01% to 3% v/v and MBCs from <0.01% to 6% v/v (Table 1). These findings are consistent with previous studies, using EOs from the same plants from which our blends were derived and tested against the same species of bacteria, but different strains to those tested in our study (Rokbeni et al., 2013; Salari, Amine, Shirazi, Hafezi, & Mohammadypour, 2006; Unlu, Ergene, Unlu, Zeytinoglu, & Vural, 2010; Wang, Li, Luo, Zu, & Efferth, 2012). Blends AB1 and AB2 were effective against antibiotic‐resistant strains *Pseudomonas aeruginosa* CIP 103467, *Staphylococcus aureus* MRSA ATCC 3359, and *Escherichia coli* ESBL (Table 1). However, *P. aeruginosa* CIP 103467 was the least sensitive to the blends tested (MBC: 3% v/v for AB2 and 6% v/v for AB1). This result was not surprising as the natural resistance of *P. aeruginosa* has been previously reported (Longbottom, Carson, Hammer, Mee, & Riley, 2004; Papadopoulos, Carson, Chang, & Riley, 2008). A combination of mechanisms protects this bacteria. The external membrane is particularly impermeable to drugs and has porine‐dependent inhibition and efflux mechanisms (Papadopoulos et al., 2008). *P. aeruginosa* employs a multidrug efflux system that extrudes compounds such as 1,8‐cineole, a monoterpene found in high levels in our blends (>40% for *R. officinalis* EO and >80% in *E. globulus* EO; see Supporting information).

**Table 1:** Minimal inhibitory concentrations (MICs), minimal bactericidal concentrations (MBCs), and MBC/MIC ratio for blends AB1 and AB2 (% v/v)

Strains	MIC		MBC		MBC/MIC ratio	
	AB1	AB2	AB1	AB2	AB1	AB2
Gram‐positive						
*Staphylococcus aureus MRSA* ATCC 33591^a^	0.38	0.38	0.38	0.75	1.00	2.00
*Streptococcus pyogenes* CIP 5641T	0.09	0.19	0.19	0.19	2.00	1.00
*Streptococcus pneumoniae* CIP 104471	0.38	0.05	0.38	0.19	1.00	4.00
*Listeria monocytogenes* CIP82110T	0.38	0.38	0.38	0.38	1.00	1.00
Gram‐negative						
*Pseudomonas aeruginosa* CIP 103467^a^	3.00	3.00	6.00	3.00	2.00	1.00
*Proteus mirabilis* CIP 103181T	0.75	0.38	0.75	0.38	1.00	1.00
*Escherichia coli ESBL* Clinical^a^	0.75	0.38	1.50	0.75	2.00	2.00
*Escherichia coli uropathogen* UTI 89	0.38	0.38	1.50	1.50	4.00	4.00
*Klebsiella pneumoniae* CIP 8291T	0.38	0.38	0.75	0.75	2.00	2.00
*Salmonella typhimurium* CIP 6062T	0.75	0.75	3.00	0.75	4.00	1.00
*Yersinia enterocolitica* CIP 8027T	0.09	0.02	0.38	0.05	4.00	2.50
*Bacteriodes fragilis* ATCC 25285	0.01	0.02	0.01	0.09	1.00	4.50
*Haemophilus influenza* IP 102514	–	0.09	–	0.09	–	1.00
*Branhamella catarrhalis* CIP 7321T	–	<0.01	–	<0.01	–	1.00

a Strain with resistance to antibiotics.

Among the Gram‐positive bacteria, AB1 and AB2 both showed the lowest MBC against *S. pyogenes* (0.19% for AB1 and 0.02% v/v for AB2). For Gram‐negative bacteria, AB1 showed the lowest MBC against *B. fragilis* (MBC: 0.01% v/v) and AB2 against *B. catarrhalis* (MBC: < 0.01% v/v; Table 1). We observed no marked differences in terms of sensitivity between Gram‐positive and Gram‐negative bacteria, results that could be attributable to a combined effect of the EOs or of some of their components. Results from the literature are conflicting. Gram‐negative bacteria were reported to be more sensitive to individual EOs (Kim, Marshall, & Wei, 1995). However, other studies found EOs were more effective against Gram‐positive bacteria or a lack of selectivity for certain EOs (Hammer, Carson, & Riley, 1999; Prabuseenivasan, Jayakumar, & Ignacimuthu, 2006).

On the basis of MBC/MIC ratios, the bactericidal effect was confirmed for AB1 and AB2 for most strains tested (ratios ≤ 2) except for *E. coli* UTI89 and *Y. enterocolitica* for the two blends, *S. thyphimurium* for AB1 and *S. pneumoniae* and *B. fragilis* for AB2 (Table 1). Discrepancies between blends may be explained by the different chemical composition of the two different cinnamon EOs. Although chemotypes of the two cinnamon EOs were the same (CT cinnamadehyde), the cinnamaldehyde concentration in the cinnamon EO was almost twofold higher in AB2 than in AB1 and the eugenol concentration was >30% in AB1 compared to ~2% in AB2.

### 3.2 Antifungal effect of AF

Blend AF had fungistatic and fungicidal activities against all *Candida* strains tested with MICs ranging from 0.01% to 0.05% v/v and minimal fungicidal concentrations (MFCs) from 0.02% to 0.05% v/v (Table 2). The MFC/MIC ratio was ≤2 for all strains tested (without specific resistance to common antifungal drugs). These results are consistent with other studies showing that EOs from *C. zeylanicum*,* E. caryophyllus*, and *O. vulgare* and their main compounds (cinnamaldehyde, eugenol, and carvacrol) were fungicidal against *C. albicans* and other *Candida* species whether or not they were resistant to common antifungal drugs (fluconazole or amphotericin B; Tampieri et al., 2005; Pinto et al., 2006; Khosravi et al., 2011; Shreaz et al., 2011).

**Table 2:** Minimal inhibitory concentrations (MICs), minimal fungicidal concentrations (MFCs) and MFC/MIC ratio for blend AF (% v/v)

Strains	MIC	MFC	MFC/MIC ratio
*Candida albicans* DSM1386	0.02	0.02	1.00
*Candida albicans* F26	0.02	0.02	1.00
*Candida albicans* F35	0.02	0.02	1.00
*Candida albicans* F78	0.02	0.02	1.00
*Candida tropicalis* IP 2148.93	0.01	0.02	2.00
*Candida glabatra* DSM 11226	0.05	0.05	1.00

### 3.3 Antiviral activity of AB1

Blend AB1 significantly reduced viral units for H1N1 and HSV1. For H1N1, a reduction greater than 99% (>2 log) was observed with 1% AB1 with a 60‐min contact time and a reduction greater than 99.99% (>4 log) with 80% and 40% AB1 after 60 min. For HSV1, a reduction greater than 99% was obtained with 1% and 40% AB1 after 60‐min contact time and a 99.99% reduction at 80% AB1 for 60 min. These results are consistent with previous work, which showed that *E. globulus* and *C. zeylanicum* EOs had antiviral activity on H1N1 and HSV1 (Astani et al., 2010; Vimalanathan & Hudson, 2014). For example, eucalyptus EO and its compounds 1,8 cineole and β‐caryophyllene exhibit an anti‐HSV1 activity by directly inactivating free‐virus particles and might interfere with virion envelope structures required for entry into host cells (Astani, Reichling, & Schnitzler, 2011; Astani et al., 2010). Commonly used antiviral medication (e.g., acyclovir and ganciclovir) inhibit DNA polymerases. Identifying substances with viral targets other than DNA polymerases are of particular interest to avoid resistance.

In a previous published study performed with a proprietary blend of rosemary, orange, clove, cinnamon, and eucalyptus EOs (On guard Wild^™^), efficacy was shown against H1N1, but was not tested against bacteria (Wu et al., 2010). In our study, AB1 was proven to be effective against both viruses and bacteria in particular, H1N1 virus, *S. aureus* and *S. pneumoniae*, two bacteria responsible for postinfluenza pneumonia (Chung & Huh, 2015). This dual activity could be of particular interest to treat influenza and also postinfluenza bacterial pneumonia infections, a leading cause of influenza‐associated death.

This in vitro study shows that blends AB1 and AB2 of *C. zeylanicum*,* D. carota*,* E. globulus*, and *R. officinalis* EOs possess a highly antimicrobial activity against Gram‐positive and Gram‐negative bacteria. Blend AB1 is also effective against viruses. Blend AF‐containing *C. zeylanicum*,* D. carota*,* S. aromaticum*, and *O. vulgare* EOs had a highly antifungal activity. This suggests that these blends could be effective to combat microorganisms involved in common, acute, and chronic human infections. Further exploration in clinical settings will be needed to confirm these in vitro results in terms of efficacy and also assess their safety.

## Conflict of Interest

Amandine Brochot and Angèle Guilbot are respectively, Project manager and Manager of the Scientific department at PiLeJe. Laïla Haddioui and Christine Roques from the Fonderephar Laboratory performed the study for PiLeJe.
