# Supplementary material for: Antibacterial, antifungal, and antiviral effects of three essential oil blends
Source: Microbiologyopen. 2017 Mar 14;6(4):e00459. doi: 10.1002/mbo3.459 (PMC5552930; doi:10.1002/mbo3.459)
Supplement: Supplementary file 1 [file MBO3-6-na-s001.docx]

**Supporting information**

***Oil extraction and composition***

Essential oils were extracted by low-pressure hydrodistillation without chemical descalers. Analyses were carried out by gas chromatography (GC) using a Hewlett-Packard CGD system (HP6850) equipped with a DB-WAX capillary column (length: 20 m; internal diameter: 100 µm; film thickness: 0.2 µm). Hydrogen was the carrier gas at a flow rate of 0.7 mL/min. Oven temperature was initially at 60°C held for 2 minutes, then gradually increased to 248°C at a 12°C/min rate and held for 2 minutes. For *E. globulus*, the column used was a DB-1 capillary column (length: 20 m; internal diameter: 100 µm; film thickness: 0.1 µm); carrier gas was the same. Oven temperature was initially at 80°C held for 2 minutes, then gradually increased to 295°C at a 12°C/min rate and held for 10 minutes. For *C. zeylanicum* provided by Bontoux, analyses were carried out by gas chromatography (GC) using a Perkin-Helmer XL system equipped with a DB-1 and a HP INNOWAX capillary column (length: 60 m; internal diameter: 250 µm; film thickness: 0.25 µm); carrier gas was the same. Oven temperature was initially at 65°C held for 2 minutes, then gradually increased to 185°C at a 2°C/min rate and then increased to 230°C and held for 11 minutes. For all extracts, samples of 0.2 µL were injected split less. Components were identified based on the comparison of their relative retention time and mass sprectra with those of laboratory bank and standard of the main components. Percentages were calculated from the area under the peak by GC/flame ionization detector. *Camelina sativa* oil was obtained by cold pressing and was then refined. The composition of essential oils is provided in Table S1.

**Table S1** Composition (%) of individual essential oils

|  | *C. zeylanicum B.** | *C. zeylanicum G.* †‡ | *Daucus carota**†‡ | *Rosmarinus officinalis**† | *Eucalyptus globulus**† | *Syzygium aromaticum*‡ | *Origanum vulgare*‡ |
| --- | --- | --- | --- | --- | --- | --- | --- |
| Isovaleraldehyde | - | - | - | - | < 0.05 | - | 2.74 |
| Tricyclene | - | - | - | 0.14 | - | - |  |
| α-pinene | - | 1.38 | 13.97 | **10.93** | 1.39 | - | - |
| α-thujene |  |  | - | 0.4 | - | - | - |
| α-fenchene | - | - | - | 0.05 | - | - | - |
| Camphene | 0.79 | 0.56 | 0.3 | 4.31 | < 0.05 | - | 0.2 |
| β-pinene | - | 0.36 | 2.89 | 8.33 | 0.3 | - | 0.23 |
| Sabinene | - | < 0.05 | 8.1 | 0.17 | - | - | < 0.05 |
| d 3 carene | - | - | - | 0.31 | - | - | - |
| Myrcene | - | 0.14 | 0.28 | 1.43 | 0.38 | - | 2.35 |
| α-phellandrene | 2.61 | 1.12 | - | 0.22 | 0.54 | - | 0.25 |
| α-terpinene | 2.13 | 1.13 | < 0.05 | 0.52 | 0.39 | - | 1.33 |
| D-Limonene | 0.91 | 1.13 | 0.23 | 2.29 | **9.01** | - | 0.29 |
| β-phellandrene | 1.88 | 4.15 | < 0.05 | - | - | - | 0.25 |
| 1,8-cineole | - | 0.19 | - | **44.52** | **83.78** | - | < 0.05 |
| cis β-ocimene | - | 0.1 | - | 0.07 | - | - | < 0.06 |
| g-terpinene | - | 0.2 | < 0.05 | 0.94 | 0.55 | - | 6.76 |
| Octanone 3 | - | 0.3 | - | 0.05 | - | - | 0.29 |
| para-cymene | 2.14 | 2.09 | < 0.05 | 0.93 | 3.58 | - | 7.71 |
| Terpinolene | - | 0.13 | < 0.05 | 0.48 | < 0.05 | - | 0.09 |
| Daucene | - | - | 1.89 | - | - | - | - |
| para-dimethylstyrene | - | - | - | - | < 0.05 | - | - |
| Pinocarvone | - | - | - | - | < 0.05 | - | - |
| Octen-1-ol-3 | - | - | - | 0.09 | - | - | 0.6 |
| trans-thujanol-4 | - |  | - | - | - |  | 0.76 |
| α-copaene | - | 0.17 | - | 0.22 | - | - | - |
| Camphor | - | 0.09 | - | **11.48** | - | - | < 0.05 |
| Linalool | 5.85 | 1.74 | < 0.05 | 0.69 | - | - | 0.19 |
| cis-α-bergamotene | - |  | - | - | - | - | - |
| trans-α-bergamotene | - |  | - | - | - | - | - |
| Bornyl acetate | - | - | - | 0.88 | - | - | < 0.05 |
| Terpinen-4-ol | - | 0.81 | 0.07 | 0.73 | < 0.05 | - | 0.62 |
| Aromadrene | - | - | - | - | < 0.05 | - | - |
| trans-pinocarveol | - | - | - | - | < 0.05 | - | - |
| Alloaromadendrene | - | - | - | - | < 0.05 | - | - |
| Furfural | - | - | - | - | - | 0.06 | - |
| β-caryophyllene | 3.58 | 2.12 | 8.96 | 3.89 | - | 4.28 | 3.22 |
| cis +trans-b-farnesene | - | - | 1.44 | - | - | - | - |
| β-bisabolene | - |  | - | - | - | - | - |
| d-terpineol | - | - | - | 0.33 | - | - | - |
| α-humulene | - | 0.54 | - | 0.38 | - | 0.51 | 0.26 |
| α-terpineol | - | 0.66 | - | 1.53 | < 0.05 | - | 0.12 |
| safrole | 1.41 | < 0.05 | - | - | - | - | - |
| trans-cinnamaldehyde | **37.55** | **65.28** | - | - | - | - | - |
| Benaldehyde | 0.35 | - | - | - | - | - | - |
| Terpenyl acetate | - | - | - | - | < 0.05 | - | - |
| Cinnamyl acetate | - | 7.4 | - |  |  | - | - |
| Ledene | - | - | - | - | < 0.05 | - | - |
| Methyl salicilate | - | - | - | - | - | 0.25 | - |
| Myrtenol | - | - | - | - | < 0.05 | - | - |
| Geraniol | - | - | - | - | < 0.05 | - | - |
| Globulol | - | - | - | - | < 0.05 | - | - |
| Methyleugenol | - | - | - | - | - | 0.03 | - |
| Eugenol | **31.64** | 2.24 | - | - | - | **81.95** | - |
| Borneol | - | - | - | 2.97 | - | - | 0.35 |
| Cinnamic alcohol | 0.05 | 0.77 | - | - | - | - | - |
| Eugenyl acetate | 1.09 | - | - | - | - | **12.13** | - |
| Geranyl acetate | - | - | - | - | - | - | 0.27 |
| β-sesquiphellandrene | - | - | - |  | - | - | - |
| Chavicol | - | - | - | - | - | 0.29 | - |
| Verbenone | - | - | - | < 0.05 | - | - | - |
| Germacrene d | - | - | - | 0.15 | - | - | < 0.05 |
| Cadinene | - | - | - | - | - | - | 0.07 |
| δ-cadinene | - | - | - | 0.22 | - | - | - |
| γ-cadinene | - | - | - | 0.10 | - | - | - |
| Caryophyllene oxyde | - | - | 1.58 | 0.07 | - | 0.24 | 0.32 |
| 2-methoxy-cinnamaldehyde | - | 0.53 | - | - | - | - | - |
| Benzyl benzoate | - | 0.73 | - | - | - | - | - |
| Thymol | - | - | - | - | - | - | 5.52 |
| Carvacrol | - | - | - | - | - | - | **63.92** |
| Geraniol | - | - | 0.27 | - | - | - | - |
| Carotol | - | - | **32.81** | - | - | - | - |
| Daucol | - | - | 1.76 | - | - | - | - |

Figures in bold indicate the five principal components. Cinnamomum zeylanicum B. provided by Bontoux. Cinnamomum zeylanicum G. provided by Golgemma. *Blend A1; † Blend A2; ‡ Blend AF
